# A Promising Tool in Retina Regeneration: Current Perspectives and Challenges When Using Mesenchymal Progenitor Stem Cells in Veterinary and Human Ophthalmological Applications

**DOI:** 10.1007/s12015-017-9750-4

**Published:** 2017-06-22

**Authors:** Anna Cislo-Pakuluk, Krzysztof Marycz

**Affiliations:** 1 Veterinary Clinic, Trzebnicka”, Kościuszki 18, 55-100 Trzebnica, Poland; 2 Department of Experimental Biology, Wrocław University of Environmental and Life Sciences, C. K. Norwida 25, 50-375 Wrocław, Poland

**Keywords:** Human retinal diseases, Glaucoma neuroprotection, Stem cell therapy, Retinal vascular diseases retinal pigment epithelium regeneration

## Abstract

Visual impairment is a common ailment of the current world population, with more exposure to CCD screens and fluorescent lighting, approximately 285 billion people suffer from this deficiency and 13% of those are considered clinically blind. More common causes for visual impairment include age-related macular degeneration (AMD), glaucoma and diabetic retinopathy (Zhu et al. *Molecular Medicine Reports*, 2015; Kolb et al. 2007; Machalińska et al. *Current Eye Research, 34*(9),748–760, 2009) among a few. As cases of retinal and optic nerve diseases rise, it is vital to find a treatment, which has led to investigation of the therapeutic potential of various stem cells types (Bull et al. *Investigative Opthalmology & Visual Science, 50*(9), 4244, 2009; Bull et al. *Investigative Opthalmology & Visual Science, 49*(8), 3449, 2008; Yu et al. *Biochemical and Biophysical Research Communications, 344*(4), 1071-1079, 2006; Na et al. *Graefe's Archive for Clinical and Experimental Ophthalmology, 247*(4), 503-514, 2008). In previous studies, some of the stem cell variants used include human Muller SCs and bone marrow derived SCs. Some of the regenerative potential characteristics of mesenchymal progenitor stem cells (MSCs) include their multilineage differentiation potential, their immunomodulatory effects, their high proliferative activity, they can be easily cultured in vitro, and finally their potential to synthesize and secrete membrane derived vesicles rich in growth factors, mRNA and miRNA which possibly aid in regulation of tissue damage regeneration. These facts alone, explain why MSCs are so widely used in clinical trials, 350 up to date (Switonski, *Reproductive Biology, 14*(1), 44–50, 2014). Animal studies have demonstrated that sub-retinal transplantation of MSCs delays retinal degeneration and preserves retinal function through trophic response (Inoue et al. *Experimental Eye Research, 85*(2), 234–241, 2007). Umbilical cord derived MSCs (UC/MSCs) have also been shown to contain neuroprotective features of ganglion cells in rat studies (Zwart et al. *Experimental Neurology, 216*(2), 439–448, 2009). This review aims to present current MSC therapies in practice, as well as their retinal regeneration potential in animal models, and their innovative prospects for treatment of human retinal diseases.

## Introduction

### Retinal Disorders in Dogs and Humans

The retina is a soft and semitransparent tissue that contains rhodopsin. It consists of an outer-pigmented layer and a neurosensory retina comprising nine layers. There are many disorders involved with the retinal pigment epithelium, the neurosensory retina and even some that affect both, these ailments are generally categorized into two paths, either acquired or congenital. Keeping these facts in mind, it was determined through thorough study that the similarities between species like dog and human are incredibly significant for disorders related to cellular and vascular changes. These two aspects, cellular and vascular, were then treated using MSC therapy, to show regeneration and protection is possible.

Congenital retinal abnormalities for dogs are either classified as hereditary or non-hereditary, although most aberrations observed to date are inherited. Inherited retinal degeneration primarily affects the photoreceptor layer, the retina and pigment epithelium (RPE) or both. Progressive retinal atrophy (PRA) is a collective term that describes a multitude of inherited retinal degenerations observed in dogs and cats. PRA is often considered to be the canine equivalent of retinitis pigmentosa (RP) which occurs in humans [11]. Interestingly this group of inherited retinal degenerations is diverse and affect a congregation of genes, they do not follow a single model of inheritance, but have many variations, and the age of onset ranges greatly. PRA has been documented in more than 100 dog breeds; this is the reason why PRA is subdivided into developmental or degenerative disease. Developmental disorders are expressed cytologically during the postnatal period where visual cell differentiation begins. These developmental disorders are characteristic of dysplasia in the rod or cone photoreceptor cells, sometimes even jointly. The rate of progression of photoreceptor loss is extremely rapid and inevitably leads to blindness. In contrast, degenerative diseases are commonly defined by defects in normally developed and differentiated photoreceptor cells. Although photoreceptor dysplasia and degenerative disease can be further subdivided, there are some common ophthalmic and clinical observations that manifest in a similar fashion. Some of the ophthalmic observations include tapetal hyperreflectivity, vascular attenuation, the optic disc becomes pale and obscured, pupillary light reflex (PLR) becomes sluggish, it’s generally bilateral and almost always leads to blindness. The most common clinical sign is impaired vision in dim light or darkness at first and then eventually difficulty seeing in bright light. Let’s consider some resemblances in human and canine disorders, for example cone degeneration (cd), an autosomal recessive canine disease causing hemeralopia, which occurs naturally in the Alaskan Malamute and German Shorthaired Pointer breeds, is phenotypically similar to human achromatopsia, a heterogenous autosomal recessive disorder [12]. Another example is congential stationary night blindness (CSNB), which is part of a group of inherited rhetinopathies of RPE, in Briard dogs is complimentary to Leber amaurosis in children [8].

Age-related macular degeneration (AMD), glaucoma and diabetic retinopathy are distinguished by the progressive loss of photoreceptors, interneurons, and retinal ganglion cells (RGCs) or RPE. Retinitis pigmentosa, Stargardt disease, Best disease, and Leber congenital amaurosis all involve early loss of photoreceptors and RGCs. While in non-mammalian vertebrates, for example amphibians or fish, after a retinal injury, the regenerative response is activated, contrastingly in humans there appears to be little to no recovery of retinal cells [13].

It was shown that injecting bone marrow mesenchymal stem cells (BMSCs) intravitreally could not only reduce damage of retinal ganglion cells (RGCs) [14] but BMSCs were also capable of mobilizing into areas where cells were subjected to laser induced injury and differentiate into retinal pigment epithelium, endothelial, pericyte and photoreceptor cells [7]. BMSCs were also observed to prolong photoreceptor survival in rhodopsin knockout mice, which could be a beneficial outcome in treatment for retinitis pigmentosa in humans [15]. Several studies have produced promising outcomes, for example BMSCs have been shown to differentiate into cells expressing photoreceptor proteins when injected into the sub-retinal space [16, 17], MSCs can express photopigment in vitro, simply by adding epidermal growth factor to the culture media [18], BMSCs or adipose derived mesenchymal stromal stem cells (ASCs) have been shown to possess the ability to differentiate into RPE cells [18, 19], to name a few. In an ischemic retina rodent model, MSCs injected into the vitrous cavity exhibited the ability to mature and secrete cilliary neurotrophic factor (CNTF), basic fibroblast growth factor (bFGF) and brain-derived neurotrophic factor (BDNF) for at least 4 weeks [20]. These results suggest that MSCs could potentially support retinal function in canine retinal pathologies, included in these are congenital, hereditary and acquired conditions, where retinal destruction is caused by various factors, for example increased intraocular pressure (IOP).

### Glaucoma

Glaucoma is a neurodegenerative disease of the retina where the main pathology is ganglion cell loss. From a cellular therapy point of view, MSCs seem to show promising results in neurodegenerative diseases [10, 21–26], they show effecient production of neurotrophic factors that support neuronal cell survival, they induce endogenous cell proliferation and promote nerve fibre regeneration at the site of injury [27]. Moreover, both human and rat MSCs, could differentiate toward neurotrophic factor secreting mesenchymal stem cells (NTF-SCs) to deliver neurotrophic factors, however NTF-SCs produce and secrete higher levels of BDNF, glial cell-derived neurotrophic factor (GDNF) and vascular endothelial growth factor (VEGF) than MSCs [27]. Intriguingly, human NTF-SCs achieved better neuroprotective effects compared with rat NTF-SCs. This evidence suggests that it may be more advantageous to use stem cells as a vector for delivery and secretion of neurotrophic factors [27]. In the ocular anterior chamber in a rat model of ocular hypertension (OHT), the application of MSCs provided neuroprotective effects in glaucoma pathophysiology via trabecular meshwork (TM) cell protection. These results demonstrate that MSCs could feasibly be a favorable tool in the treatment of ocular hypertension and retinal cell degeneration [28]. The effect of MSC transplantation in vivo, demonstrated to be rapid and long lasting in significant reduction of IOP induced by episcleral veins (EVC) in eyes with hypertension. MSCs seemed to be associated with ciliary processes and the trabecular meshwork. Quantification of RGCs on whole flat-mounted retina established a protective effect of MSCs on their viability. In the same study it was noted that MSCs in conditioned medium in viro promote the following: (i) human trabecular meshwork (hTM) cells survive by activation of the antiapoptotic pathway, Akt; (ii) hTM cells relax as myosin phosphorylation decreased; (iii) hTM cells do not acquire transforming growth factor-β2-dependent profibrotic phenotype. In conclusion the proregenerative effects of MSCs in glaucoma could be allocated to production and delivery of neurotrophic factors, to influence on ganglion cells to have antiapoptotic characteristics and to trabecular meshwork protection [28].

### Protocols for MSC Administration

The study conducted by Johnson TV, Bull ND, Hunt DP et al. revealed that transplantation of BM-MSCs into the vitrous body of rats with glaucoma resulted in a 30% increase of optic nerve axonal survival [29]. Beyond intravitreal injection, MSCs were delivered according to different pathways as well. It was well recognized that systematically administered GFP-marked MSCs may be incorporated into the neuroretinal tissues and play an important role in wound modulation of physically damaged retinal tissue [1]. Using intravenous MSCs as adjuvant therapy with sub-retinal injection might improve cell therapy treatments for retinal dystrophy [30]. Recently the Royal College of Surgeons (RCS) studied safety and efficacy of sub-retinal injection of human Wharton’s Jelly-derived mesenchymal stem cells (hWJ-MSCs) on retinal structure and function in rats. Sub-retinal injection of hWJ-MSCs delayed the loss of ONL in RCS rats. Although hWJ-MSCs show potential to differentiate into retinal-like cells and appear to be safe in nature, this type of cell-based therapeutic treatment for retinal dystrophies still warrants more research. Administration of MSCs in close proximity to the retina also proved to be effective in treating retinal degeneration in the RCS rats by transplanting hBM-MSCs in a thin epiretinal layer [31]. This method of transplantation proximally to the retina actually proved more effective than intravitreal injection [31].

### Immune Reaction in Graft-Host Integration

Most studies confirmed limited graft-host integration, although there were a few that managed to demonstrate successful integration of stem cells to the retina [32]. One of the main advantages of MSC and NTF-SC therapy is the possibility for autologous transplantation using the patient’s own bone marrow-derived stem cells. This approach solves the problem with immune rejection, does not lead to the formation of teratomas and is free of ethical and or political concerns.

### New System for Cell Transplantation

Recently, direct injections methods, using innovative syringe devices, have been employed for cell-based therapy for treatment of retinal degeneration. [33]. In rat model, bone marrow mesenchymal stem cells (BMSCs) were successfully transplanted using syringe with flexible needle and adjustable pin without the use of additive immunosuppressive therapy [33].

### Retinal Vascular Diseases

For diabetic retinopathy or retinal vein occlusion successful therapy was reported when mesenchymal stem cells or autologous bone marrow CD44+ cells were used. Moreover, adipose stromal cells or pluripotent stem cells were considered as potential therapeutic agents in retinal vascular disorders [34]. In clinical studies, it was shown that both endothelial progenitor cells and adipose stromal cells are able to integrate into the damaged retinal vascular wall of patients suffering from diabetic retinopathy and ischemia-reperfusion injury [34]. Interestingly, mesenchymal stem cells rather do appear to exert paracrine effect, but did not integrate readily to damaged retinal wall. In the context of safeness and efficacy, various stem cells therapies for treating retinal vascular disorders require further investigation [34].

### Retinal Pigment Epithelium Regeneration

Although RPE cell and MSCs derived from different germ layers, MSCs are able to act via mesodermal differentiation. Huag et al. showed that RPE-like cells could differentiate from MSCs by culture them with RPE conditional medium supplemented with POS [4]. Other studies showed that MSCs are able to differentiate into retinal cells and substitute the damaged retinal cells under certain conditions [13, 35, 36]. Moreover, it was showed that RPE cells could differentiate from MSCs using anti-miR-410 treatment without use of additional factors [37].

Obtained cells have similar morphology and phagocytic capabilities to native RPE cells. Clinical studies indicated that after MSCs injection, RPE cells originated from MSCs could be found in the sodium iodide-damaged retina after sub-retinal with 5 days [13, 38].

### Post-Transplantation Complications

Although current MSCs therapies are the most promising treatments in ophthalmology, the restrictions are also occurring. Between others, one of the major limitation is extensive reactive gliosis production in the recipient retina after MSC injection [29, 39]. Stem cell application to patient suffer from retinal degeneration disease may achieve minimal success due to limited stem cell migration into the host tissue. This adverse reaction is caused by reactive gliosis and chondroitin sulfate proteoglycan deposition [5, 29, 40–42] which was observed in vivo after intravitreal BM-MSC transplantation [43].

### Conclusions and Perspecives for Further Development in Opthalmology

Stem cells have been investigated in opthalmological research as a forthcoming tool for retinal degeneration. Mesenchymal stem cells have exhibited many advantages because of their multilineage differentiation potential, the ease in their culturing and their immunomodulatory properties which are crucial in retinal regeneration research. Current exploration has determined new mechanisms of regeneration and MSC protective capabilities, on degeneration of different types of retinal cell ad retinal vessels. Mesenchymal stem cell-derived microvesicles (MVs) allow for developments in future research and clinical applications as a result of their availability as well as the growth factors, miRNA and mRNA they possess. Studies have shown that the application of MVs in regenerative medicine proves to be very dynamic, which is directing clinical research in opthamology towards this domain of study. In the grand scheme of scientific interest, it is expected that MVs may have higher output and potential in retinal regeneration than stem cell therapies have so far, therefore it is anticipated that this research field will be moving further into this direction.

## References

[1] Zhu Q, Liu Z, Wang C et al. (2015). Lentiviral-mediated growth-associated protein-43 modification of bone marrow mesenchymal stem cells improves traumatic optic neuropathy in rats. *Molecular Medicine Reports, 12*(4), 5691-700. 10.3892/mmr.2015.4132 PMC4581804 26238991

[2] Kolb H, Fernandez E, Nelson R (2007). Webvision.

[3] Machalińska A, Baumert B, Kuprjanowicz L, Wiszniewska B, Karczewicz D, Machaliński B (2009). Potential Application of Adult Stem Cells in Retinal Repair—Challenge for Regenerative Medicine. Current Eye Research.

[4] Bull N, Irvine K, Franklin R, Martin K (2009). Transplanted Oligodendrocyte Precursor Cells Reduce Neurodegeneration in a Model of Glaucoma. Investigative Opthalmology & Visual Science.

[5] Bull N, Limb G, Martin K (2008). Human Müller Stem Cell (MIO-M1) Transplantation in a Rat Model of Glaucoma: Survival, Differentiation, and Integration. Investigative Opthalmology & Visual Science.

[6] Yu S, Tanabe T, Dezawa M, Ishikawa H, Yoshimura N (2006). Effects of bone marrow stromal cell injection in an experimental glaucoma model. Biochemical and Biophysical Research Communications.

[7] Na L, Xiao-rong L, Jia-qin Y (2008). Effects of bone-marrow mesenchymal stem cells transplanted into vitreous cavity of rat injured by ischemia/reperfusion. Graefe's Archive for Clinical and Experimental Ophthalmology.

[8] Switonski M (2014). Dog as a model in studies on human hereditary diseases and their gene therapy. Reproductive Biology.

[9] Inoue Y, Iriyama A, Ueno S (2007). Subretinal transplantation of bone marrow mesenchymal stem cells delays retinal degeneration in the RCS rat model of retinal degeneration. Experimental Eye Research.

[10] Zwart I, Hill A, Al-Allaf F (2009). Umbilical cord blood mesenchymal stromal cells are neuroprotective and promote regeneration in a rat optic tract model. Experimental Neurology.

[11] Wiik A, Ropstad E, Ekesten B, Karlstam L, Wade C, Lingaas F (2015). Progressive retinal atrophy in Shetland sheepdog is associated with a mutation in theCNGA1gene. Animal Genetics.

[12] Sidjanin D (2002). Canine CNGB3 mutations establish cone degeneration as orthologous to the human achromatopsia locus ACHM3. Human Molecular Genetics.

[13] Gong L, Wu Q, Song B, Lu B, Zhang Y (2008). Differentiation of rat mesenchymal stem cells transplanted into the subretinal space of sodium iodate-injected rats. Clinical & Experimental Ophthalmology.

[14] Pascolini D, Mariotti S (2011). Global estimates of visual impairment: 2010. British Journal of Ophthalmology.

[15] Arnhold S, Absenger Y, Klein H, Addicks K, Schraermeyer U (2006). Transplantation of bone marrow-derived mesenchymal stem cells rescue photoreceptor cells in the dystrophic retina of the rhodopsin knockout mouse. Graefe's Archive for Clinical and Experimental Ophthalmology.

[16] Bunce C, Wormald R (2006). Leading causes of certification for blindness and partial sight in England & Wales. BMC Public Health.

[17] Castanheira P, Torquetti L, Nehemy M, Goes A (2008). Retinal incorporation and differentiation of mesenchymal stem cells intravitreally injected in the injured retina of rats. Arquivos Brasileiros de Oftalmologia.

[18] ZHANG P, Li J, LIU Y (2009). Human neural stem cell transplantation attenuates apoptosis and improves neurological functions after cerebral ischemia in rats. Acta Anaesthesiologica Scandinavica.

[19] Vossmerbaeumer U, Ohnesorge S, Kuehl S (2009). Retinal pigment epithelial phenotype induced in human adipose tissue-derived mesenchymal stromal cells. Cytotherapy.

[20] Erices A, Conget P, Minguell J (2000). Mesenchymal progenitor cells in human umbilical cord blood. British Journal of Haematology.

[21] Johnson T, Bull N, Martin K (2011). Stem cell therapy for glaucoma: possibilities and practicalities. Expert Review of Ophthalmology.

[22] Johnson T, Bull N, Martin K (2008). Transplantation prospects for the inner retina. Eye.

[23] Lebrun-Julien F, Di Polo A (2008). Molecular and Cell-Based Approaches for Neuroprotection in Glaucoma. Optometry and Vision Science.

[24] Siqueira R (2011). Stem cell therapy for retinal diseases: update. Stem Cell Research & Therapy.

[25] Miller N (2001). Optic nerve protection, regeneration, and repair in the 21st century: LVIII Edward Jackson Memorial lecture. American Journal of Ophthalmology.

[26] Ofri R, Narfström K (2007). Light at the end of the tunnel? Advances in the understanding and treatment of glaucoma and inherited retinal degeneration. The Veterinary Journal.

[27] Levkovitch-Verbin H, Sadan O, Vander S (2010). Intravitreal Injections of Neurotrophic Factors Secreting Mesenchymal Stem Cells Are Neuroprotective in Rat Eyes following Optic Nerve Transection. Investigative Opthalmology & Visual Science.

[28] Roubeix C, Godefroy D, Mias C (2015). Intraocular pressure reduction and neuroprotection conferred by bone marrow-derived mesenchymal stem cells in an animal model of glaucoma. Stem Cell Research & Therapy.

[29] Johnson T, Bull N, Hunt D, Marina N, Tomarev S, Martin K (2010). Neuroprotective Effects of Intravitreal Mesenchymal Stem Cell Transplantation in Experimental Glaucoma. Investigative Opthalmology & Visual Science.

[30] Bakondi B, Girman S, Lu B, Wang S (2016). Multimodal Delivery of Isogenic Mesenchymal Stem Cells Yields Synergistic Protection from Retinal Degeneration and Vision Loss. Stem Cells Translational Medicine.

[31] Tzameret A, Sher I, Belkin M (2015). Epiretinal transplantation of human bone marrow mesenchymal stem cells rescues retinal and vision function in a rat model of retinal degeneration. Stem Cell Research.

[32] Bartsch U, Oriyakhel W, Kenna P (2008). Retinal cells integrate into the outer nuclear layer and differentiate into mature photoreceptors after subretinal transplantation into adult mice. Experimental Eye Research.

[33] Rotenstreich Y, Tzameret A, Kalish S (2015). A novel system for minimally invasive transplantation of bone marrow derived stem cells as a thin layer in the subretina and extravascular spaces of the choroid--for treatment of retinal degeneration. Harefuah.

[34] Park S. (2016) Cell Therapy Applications for Retinal Vascular Diseases: Diabetic Retinopathy and Retinal Vein Occlusion. *Investigative Opthalmology & Visual Science, 57*(5):ORSFj1. 10.1167/iovs.15-17594 27116667

[35] Tomita M, Adachi Y, Yamada H (2002). Bone Marrow-Derived Stem Cells Can Differentiate into Retinal Cells in Injured Rat Retina. Stem Cells.

[36] Marycz K, Grzesiak J, Wrzeszcz K, Golonka P (2012). Adipose stem cell combined with plasma-based implant bone tissue differentiation in vitro and in a horse with a phalanx digitalis distalis fracture: a case report. Veterinární Medicína.

[37] Choi S, Kim J, Seo M (2017). Inhibition by miR-410 facilitates direct retinal pigment epithelium differentiation of umbilical cord blood-derived mesenchymal stem cells. Journal of Veterinary Science.

[38] Dang Y, Zhang C, Zhu Y. (2015). Stem cell therapies for age-related macular degeneration: the past, present, and future. *Clinical Interventions in Aging, 10*, 255–264. 10.2147/CIA.S73705 PMC4298283 25609937

[39] Menet V, Ribotta M, Chauvet N et al. (2017). Inactivation of the Glial Fibrillary Acidic Protein Gene, But Not That of Vimentin, Improves Neuronal Survival and Neurite Growth by Modifying Adhesion Molecule Expression [Internet]. Jneurosci.org. [cited 2017 Apr 18];Available from: http://www.jneurosci.org/content/21/16/6147.short. 10.1523/JNEUROSCI.21-16-06147.2001 PMC6763158 11487638

[40] Barber A, Hippert C, Duran Y (2012). Repair of the degenerate retina by photoreceptor transplantation. Proceedings of the National Academy of Sciences.

[41] Singhal S, Lawrence J, Bhatia B (2008). Chondroitin Sulfate Proteoglycans and Microglia Prevent Migration and Integration of Grafted Müller Stem Cells into Degenerating Retina. Stem Cells.

[42] Grzesiak J, Marycz K, Wrzeszcz J, Czogala J (2011). Isolation and Morphological Characterisation of Ovine Adipose-Derived Mesenchymal Stem Cells in Culture. International Journal of Stem Cells.

[43] Tassoni A, Gutteridge A, Barber A, Osborne A, Martin K (2015). Molecular Mechanisms Mediating Retinal Reactive Gliosis Following Bone Marrow Mesenchymal Stem Cell Transplantation. Stem Cells.

